# Fabricated Gamma-Alumina-Supported Zinc Ferrite Catalyst for Solvent-Free Aerobic Oxidation of Cyclic Ethers to Lactones

**DOI:** 10.3390/molecules28207192

**Published:** 2023-10-20

**Authors:** Naaser A. Y. Abduh, Abdullah A. Al-Kahtani, Mabrook S. Amer, Tahani Saad Algarni, Abdel-Basit Al-Odayni

**Affiliations:** 1 Department of Chemistry, King Saud University, Riyadh 11451, Saudi Arabia; akahtani@ksu.edu.sa (A.A.A.-K.); msamer@ksu.edu.sa (M.S.A.);; 2 Restorative Dental Sciences Department, College of Dentistry, King Saud University, Riyadh 11545, Saudi Arabia

**Keywords:** cyclic ethers, tetrahydrofuran, supported catalyst, lactones, aerobic oxidation, oxygen

## Abstract

The aim of this work was to fabricate a new heterogeneous catalyst as zinc ferrite (ZF) supported on gamma-alumina (γ-Al_2_O_3_) for the conversion of cyclic ethers to the corresponding, more valuable lactones, using a solvent-free method and O_2_ as an oxidant. Hence, the ZF@γ-Al_2_O_3_ catalyst was prepared using a deposition–coprecipitation method, then characterized using TEM, SEM, EDS, TGA, FTIR, XRD, ICP, XPS, and BET surface area, and further applied for aerobic oxidation of cyclic ethers. The structural analysis indicated spherical, uniform ZF particles of 24 nm dispersed on the alumina support. Importantly, the incorporation of ZF into the support influenced its texture, i.e., the surface area and pore size were reduced while the pore diameter was increased. The product identification indicated lactone compound as the major product for saturated cyclic ether oxidation. For THF as a model reaction, it was found that the supported catalyst was 3.2 times more potent towards the oxidation of cyclic ethers than the unsupported one. Furthermore, the low reactivity of the six-membered ethers can be tackled by optimizing the oxidant pressure and the reaction time. In the case of unsaturated ethers, deep oxidation and polymerization reactions were competitive oxidations. Furthermore, it was found that the supported catalyst maintained good stability and catalytic activity, even after four cycles.

## 1. Introduction

Nowadays, environmental changes have triggered scientists to focus on finding solutions to reduce pollution levels from several sources [1]. One example of these is chemical industries, a factor that has an enormous effect on air and water pollution, resource exhaustion, soil contamination, and greenhouse gases [2,3,4]; there is now a significant increase in interest in searching for remedies that refer to the principles of green chemistry by which pollution is reduced to the minimal levels [5,6]. It is fundamental that catalysts, the atomic economy, harmless and sustainable materials, selective reactions, and other green chemistry principles are put into consideration when searching for unprecedented techniques in the chemical industry to substitute the non-environmental currently approved approaches [7,8].

Lactones are cyclic organic compounds of carboxylic esters that are typically categorized according to the type of rings as having three, four, five, six, and seven members, namely, α-, β-, γ-, δ-, and ω-lactones, respectively [9]. Due to their availability in nature, uses, and stability, the five- and six-membered ones are considered the most significant [10]. The most varied and well-liked gamma-lactones are represented by γ-butyrolactone (GBL) and γ-valerolactone (GVL), while δ-valerolactone (DVL) is the most popular delta-lactone.

They have numerous applications in manufacturing polymers, chemical intermediates, pharmaceuticals, foods, beverages, electrical products, rubber additives, herbicides, solvents, viscosity modifiers, textiles, agrochemicals, pesticides, and liquid fuels [11,12,13,14,15,16]. In addition, they are key components of many biological compounds and, as such, play a significant role in biology [17,18]. Hence, recently, the lactone marketing value has grown greatly [19,20].

Until now, lactone production is still based on three methods in general. The first is the oxidative lactonization of corresponding diols, e.g., 1,4 butanediol and 1,5 pentanediol, as raw materials for GBL and DVL production, respectively. The second is reductive lactonization of γ-keto acid(ester)s, e.g., levulinic acid, which is famous for GVL production. The third is the Baeyer–Villiger oxidation of cyclic ketones, which is mostly applied for DVL synthesis [21]. Moreover, the malic anhydride method is a well-known industrial route for GBL production [22]. From the scientific point of view, these methods are environmentally unsafe and economically unsatisfactory [16,23,24,25,26]. As a consequence, finding renewable, environmentally friendly, and economical alternative methods for lactone production is of utmost importance [27,28,29].

Today, biomass-derived products are of high economic and environmental importance as they, from a green chemistry point of view, represent a sustainable raw material [29,30]. It is possible to synthesize lactones from biomaterials using chemical and biological methods, but these methods need to be improved [31,32,33].

Cyclic ethers such as tetrahydrofuran (THF), tetrahydropyran (THP), and 2-methyl tetrahydrofuran (2-MTHF) can be utilized as excellent sustainable raw materials for GBL, DVL, and GVL production, respectively, for which they can be sourced from biomass-derived material [34,35,36]. Notably, cyclic ethers have similar backbone as corresponding lactones and could be oxidized in one step to lactones. However, such a process may require an appropriate catalyst that could achieve a satisfactory economic yield and is environmentally unharmful.

Several studies have been conducted to explore new environmentally friendly oxidation processes [37,38,39]. For example, Higuchi et al. applied Ru porphyrin heteroaromatic N-oxide system for homogenous cyclic ether oxidation [37]. In addition to drawbacks related to homogenous catalysts, such as production waste and economic cost [40], over-oxidation and ring opening reactions also reduce the lactone yield [41]. Tert-butyl hydroperoxide/(diacetoxyiodo) benzene (DIB/TBHP) protocol, depending on free radicals mechanism with no metallic reagent under mild condition, have been used for the oxidative transformation of cyclic ethers to lactones [38]. Although this protocol prevents the over-oxidized product, processing requirements, moisture sensitivity, necessary solvents, and complex product separation make its use on a large industrial scale unfeasible. Bhaumikb et al. [42] studied the catalytic oxidation of cyclic ethers to the corresponding lactones, employing various crystalline microporous metallosilicates as heterogeneous catalysts in the liquid phase. They used hydrogen peroxide (H_2_O_2_) as one green oxidizing agent for cyclic ether oxidation. It is, however, essential to note that acceptable catalysts should be inexpensive and easy to obtain. In general, hydrogen peroxide is a remarkably effective oxidant due to its oxidative features represented by the extra oxygen atom compared to water’s structure [43]. However, handling requirements, high cost, and product separation have limited its utilization [44]. On the other hand, despite its limited ability to oxidize under mild conditions and low selectivity of desired products, molecular oxygen, the ultimate green oxidizing agent, is a low-cost, cheap, and readily available gas [45,46]. Thus, it is desirable to overcome this limitation by finding an appropriate heterogeneous catalyst [47,48]. 

In this regard, gold (Au) supported by nanorods of CeO_2_ has been tested as a heterogeneous catalyst for cyclic ether oxidation; using THF as a model reaction, oxidation was performed under aerobic conditions to produce GBL as a main product and propylformate as a minor product [39]. Nevertheless, it is still required to improve yield and selectivity while avoiding environmental and economic issues.

In our previous article [49], we reported that the use of ZnFe_2_O_4_ catalyst and H_2_O_2_ as an oxidizing agent for THF conversion into GBL resulted in 47.3% conversion and 87.2% selectivity. When O_2_ was used in place of H_2_O_2_, a conversion and a GBL selectivity of 18.1% and 63.3%, respectively, were achieved. Indeed, such unsatisfied improvement can be further ameliorated by incorporating certain support. Hence, materials like Al_2_O_3_, SiO_2_, TiO_2_, and ZrO_2_ can be used as catalysts, composite agents, or supports for enhancing catalyst reactivity [50]. Support could play a synergistic effect to increase catalyst efficiency. γ-Al_2_O_3_ is an active catalyst and support with many attractive properties such as low cost, surface area, good thermal stability, and acid/base characteristics [51,52]. 

Herein, ZnFe_2_O_4_/γ-Al_2_O_3_-supported catalyst was successfully fabricated by a simple deposition–coprecipitation method, being further employed as an effective catalyst for cyclic ether oxidation to lactones through a green process, which involves solvent-free aerobic oxidation under mild conditions. The prepared catalyst was extensively characterized using IR, XRD, TGA, EDS, ICP, XPS, BET surface area, SEM, and TEM. The effect of various factors, including reaction temperature, reaction time, oxidant amount, and catalyst dose, were fully investigated for THF as a model reaction of cyclic ether oxidation. Moreover, oxidation of several five- and six-membered saturated and unsaturated cyclic ethers was also explored.

## 2. Results and Discussion

### 2.1. Catalyst Characterizations

#### 2.1.1. FTIR Analysis

**Figure 1:**
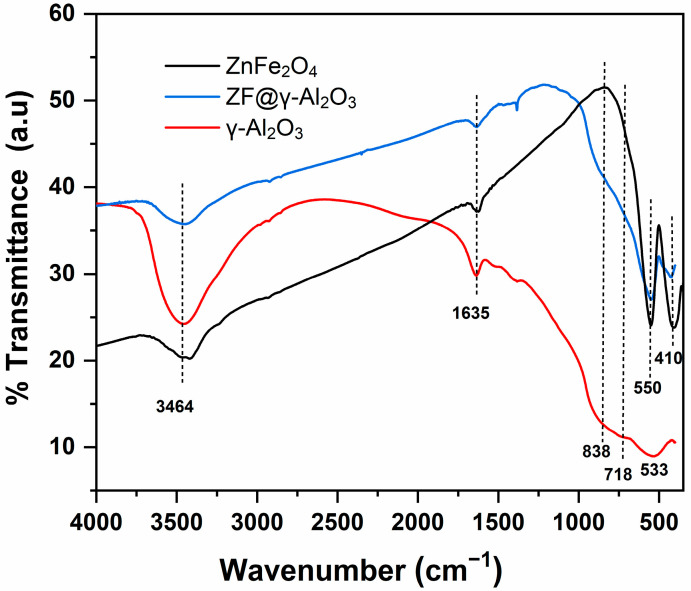
FTIR spectra of ZnFe_2_O_4_ (ZF), ZF@γ-Al_2_O_3_, and γ-Al_2_O_3._

Figure 1 shows the FTIR spectra of ZnFe_2_O_4_ (ZF), ZF@γ-Al_2_O_3_, and γ-Al_2_O_3_. In this figure, peaks of adsorbed water were observed in all spectra around 3460 and 1635 cm^−1^ for stretching and bending vibrations, respectively. The characteristic broad peaks at 838, 757, and 533 cm^−1^ belong to the Al–O–Al vibration of the γ-Al_2_O_3_ support [53]. Moreover, the spectrum of the ZF spinal catalyst showed two peaks at 550 and 410 cm^−1^, which are attributed to Zn–O and Fe–O vibrations at tetrahedral and octahedral sites, respectively [54]. Moreover, the shoulder-type peaks at 864 and 718 cm^−1^, as well as peaks at 543 and 427 cm^−1^, confirm the formation of the ZF@γ-Al_2_O_3_-supported catalyst composite.

#### 2.1.2. XRD Analysis

**Figure 2:**
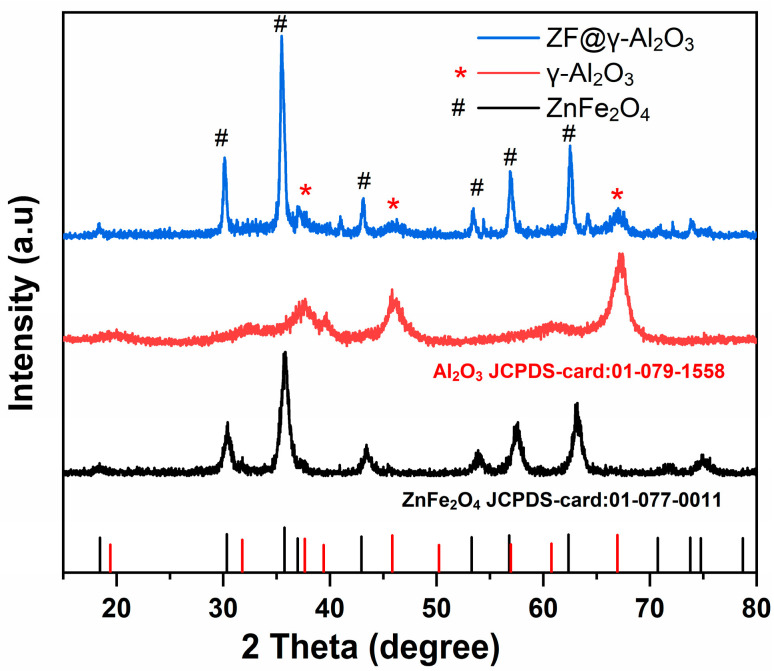
XRD spectra of ZnFe_2_O_4_ (ZF), ZF@γ-Al_2_O_3_, and γ-Al_2_O_3_ with JCPDS card.

The X-ray diffractograms of ZF, ZF@γ-Al_2_O_3_, and γ-Al_2_O_3_ are illustrated in Figure 2. The XRD pattern of zinc ferrite with a cubic spinel crystal structure showed a list of 2θ peaks at 18.45°, 30.42°, 35.65°, 43.53°, 53.87°, 57.61°, 63.19°, 71.75°, 74.79°, and 78.82°, which agreed with the reported JCPDS PDF (card no: 01-077-0011) [55]. The typical bands of γ-Al_2_O_3_ were observed at 31.10°, 37.69°, 39.67°, 45.94°, 60.96°, and 67.40° [56], which matched the diffraction planes (220), (311), (222), (400), (511), and (440) in the JCPDS PDF (card no: 01-079-1558), respectively. The XRD pattern of ZF@γ-Al_2_O_3_ indicated, besides the peaks of ZF, the presence of Al_2_O_3_ prominent peaks at 37.67°, 45.92°, and 67.37°, thus signifying the composite formation. Indeed, the XRD pattern of the catalyst was in good agreement with the literature [57,58].

The Debye–Scherrer formula was used to calculate the average crystal size of ZF particles in the supported catalyst, which amounted to 18.86 nm. Moreover, the unit constant was also measured, and the result was 8.31, approving the formation of a cubic spinel structure [59].

#### 2.1.3. TGA Analysis

**Figure 3:**
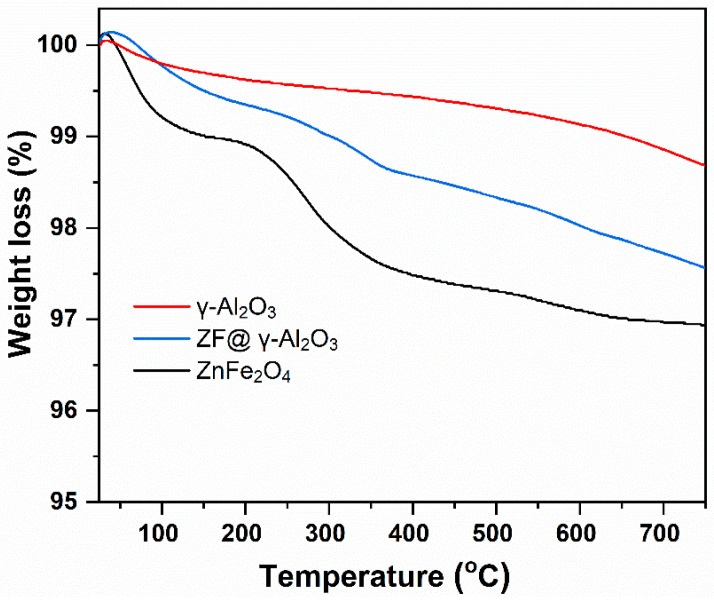
TGA curves of ZnFe_2_O_4_ (ZF), γ-Al_2_O_3_, and ZF@γ-Al_2_O_3._

The thermal stabilities of the target substances, the catalyst ZF, the support γ-Al_2_O_3_, and the applied γ-Al_2_O_3_-supported ZF (ZF@γ-Al_2_O_3_) catalyst were monitored using the TGA technique. As can be seen, three degradation steps can be identified in Figure 3. Two weight-loss stages are presented for the ZF catalyst ranging from 25 to 400 °C, with a mass loss of 1.2 and 1.4%, attributed to the loss of physically adsorbed water and lattice water molecule evaporation, respectively [60,61]. However, a mass loss of 0.5% for γ-Al_2_O_3_ and 1.43% in the case of ZF@γ-Al_2_O_3_ indicated the effect of the amount of γ-Al_2_O_3_ in the composite. Moreover, this result confirmed that a stable composite was successfully formed in the calcined catalyst up to 800 °C as there was no significant weight loss.

#### 2.1.4. BET-Surface Area Analysis

**Figure 4:**
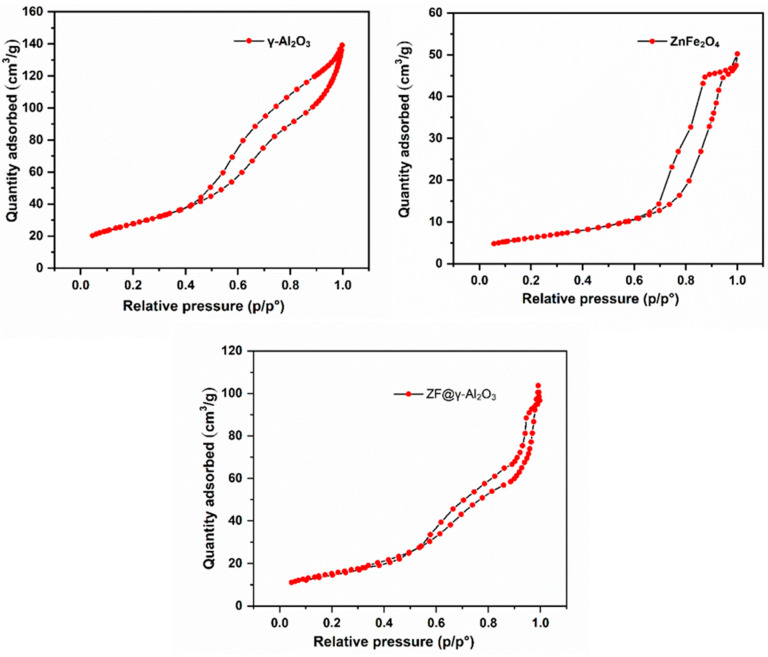
Nitrogen adsorption–desorption isotherms of γ-Al_2_O_3_, ZnFe_2_O_4_ (ZF), and ZF@γ-Al_2_O_3._

The BET-surface area, pore volume, and pore width of the unsupported and supported catalysts were measured using N_2_ adsorption–desorption isotherms and the Barrett–Joyner–Halenda (BJH) method. According to IUPAC classification, Figure 4 supports the IV-type isotherms for both ZF and γ-Al_2_O_3_ and the majority of the mesoporous structure. For hysteresis loop classification, γ-Al_2_O_3_ has a type between H1 and H2 [62], whereas ZF has an H1 type. Most importantly, the change in the hysteresis shape of the supported catalyst is further evidence of incorporating ZF particles in the supported matrix. Moreover, the shift in the hysteresis loop at relative pressure (P/P°) from 0.42 for γ-Al_2_O_3_ to 0.6 for ZF@γ-Al_2_O_3_ indicates increased pore width in the supported catalyst [63]. According to Table 1, upon loading of the ZF to the γ-Al_2_O_3_ support, the latter total surface area, external surface area, and average pore volume decreased. The decrease in the surface area of support can be attributed to the dispersed ZF particles on the surface and the blockage of inner pores. In addition, the average pore width was substantially higher for ZF@γ-Al_2_O_3_ due to blocking the small pores, which were thus eliminated from pore counting [64].

#### 2.1.5. TEM Analysis

**Figure 5:**
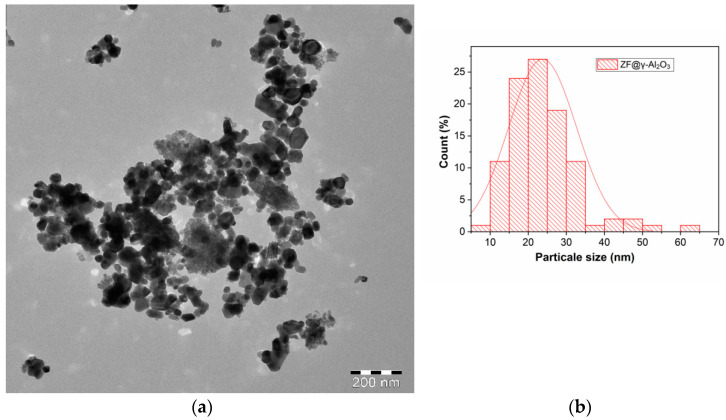
(**a**) TEM micrograph of ZF@γ-Al_2_O_3_, and (**b**) particle size distribution.

Surface morphology, shape, and particle size distribution of the obtained supported catalyst were evaluated using a transmission electron microscope (TEM). As shown in Figure 5a, the TEM micrograph confirmed the formation of the cubic ZF structure, which was highly dispersed on the γ-Al_2_O_3_ support. Based on the TEM image, particle size and particle distribution were also counted and are presented in Figure 5b. Accordingly, the catalyst particles were of irregular shapes with an average particle size of 24 nm.

#### 2.1.6. SEM Analysis

**Figure 6:**
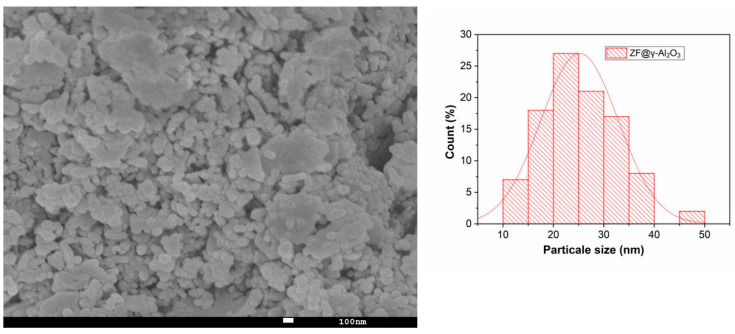
SEM images of ZF@γ-Al_2_O_3_.

The surface morphology of the supported catalyst is illustrated in Figure 6. It is shown that the particles were majorly spherical. The particle size of the dispersed ZF was counted to 26 nm. As can be seen, the obtained particle sizes from both TEM and SEM were almost the same, with further evidence of the catalyst nanostructure and catalyst-support compatibility.

#### 2.1.7. EDS Analysis

**Figure 7:**
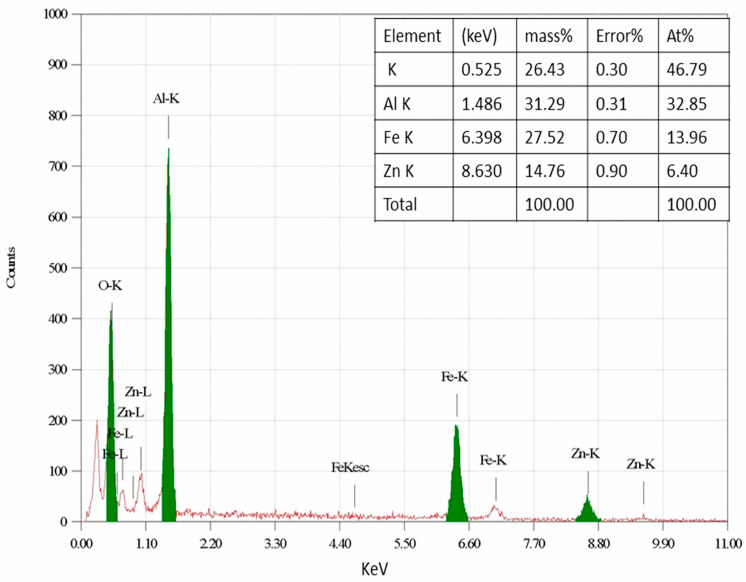
EDS of ZF@γ-Al_2_O_3_.

The chemical composition of the prepared ZF@γ-Al_2_O_3_ catalyst was investigated using the energy-dispersive X-ray spectroscopy (EDS) method, as depicted in Figure 7. The EDS spectrum shows the presence of aluminum (Al), zinc (Zn), iron (Fe), and oxygen (O) peaks, clarifying the composition of the supported catalyst. The mole percentage of the ZF catalyst in the supported catalyst was calculated to be 28%, indicating a homogeneous composition of the supported catalyst.

#### 2.1.8. XPS Analysis 

**Figure 8:**
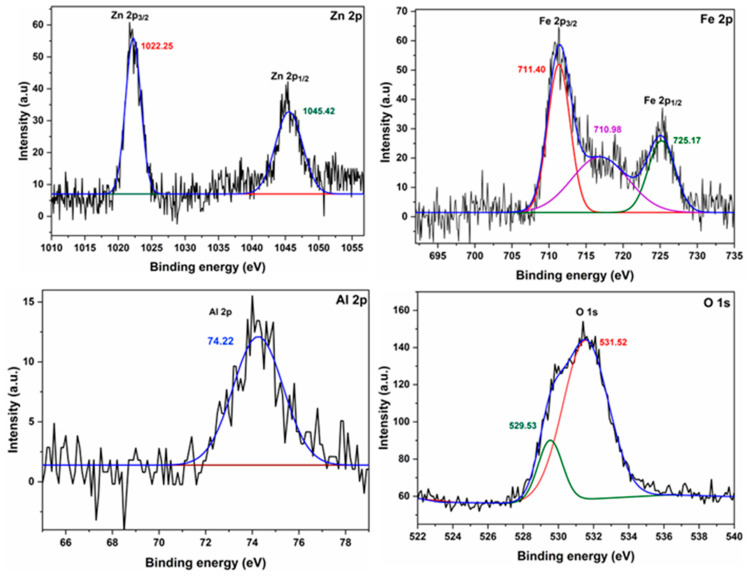
XPS spectra of ZF@γ-Al_2_O_3._

The supporting catalyst’s atomic composition was identified using X-ray photoelectron spectroscopy (XPS). Zn, Fe, Al, and O elements were detected, as shown in Figure 8. The distinctive peaks of Zn 2p_3/2_ and Zn 2p_1/2_ were found in the spectra of the ZF@γ-Al_2_O_3_ at 1022.25 eV and 1045.42 eV, respectively. The peak with characteristic binding energy 531.52 eV and 529.53 eV belongs to the O1s species. In the spectrum of Fe 2p, there were two peaks at 721.40 eV and 725.17 eV, indicating the presence of Fe^3+^. Furthermore, the peak at 74.22 eV was assigned to Al 2p. Those results agreed with the ZF@γ-Al_2_O_3_ reported spectra [57]. In addition, comparing the ZF@Al_2_O_3_ spectrum with the ZF XPS spectrum [49] (not shown), a shift in band energies of Zn 2p, Fe 2p, and O 1s was observed, which suggests that the electrical environment in the composite changed [65].

#### 2.1.9. ICP Analysis

Elemental analyses were carried out under inductively coupled plasma spectrometry (ICP) to confirm the chemical composition of the prepared supported catalyst. The results are summarized in Table 2. The molar percentages of zinc, iron, and aluminum revealed by the ICP analysis agreed with those obtained by the EDS analysis (Section 2.1.7).

### 2.2. Catalytic Activity

**Scheme 1:**
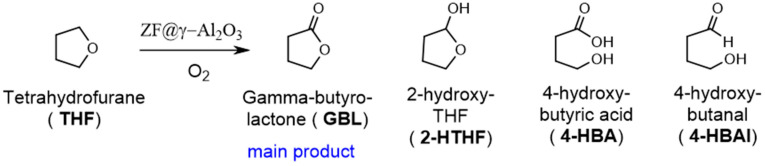
Reaction products of THF oxidation by O_2_ over the ZF@γ-Al_2_O_3_ catalyst.

The as-prepared supported catalyst was tested for aerobic oxidation of cyclic ethers. The reactions were carried out in a liquid phase without solvent. The identification of the products was achieved by GC-MS. Moreover, the products’ conversion and selectivity were determined using gas phase chromatography. THF oxidation was used as a model reaction of cyclic ether oxidation to evaluate the catalyst’s performance. The analysis showed that the reaction led to gamma-butyrolactone (GBL) as a mine product, while 2-hydroxy tetrahydrofuran (2-HTHF), 4-hydroxybutyric acid (4-HBA), and 4-hydroxybutaldehyde (4-HBAl) were the minor products (Scheme 1). Furthermore, a blank run experiment was also conducted for comparison, which revealed a negligible conversion, demonstrating that O_2_ alone cannot oxidize cyclic ethers without a catalyst. According to the preliminary tests, ZF@γ-Al_2_O_3_ showed better performance than the individuals γ-Al_2_O_3_ and ZnFe_2_O_4_ separately. This suggests a synergistic effect of catalyst and support, encouraged by the support’s high surface area and physiochemical properties. Large surface areas are advantageous for dispersing ZF as active components, while adequate pore sizes provide reactants additional sites to bind and activate gaseous oxygen species [66]. In essence, the catalytic activity of supporting catalysts depends on their acid–base property. Hence, ether is anticipated to be readily adsorbed and activated on surfaces [67,68]. 

The effect of the catalyst-support ratio was investigated for three different catalyst-support mole ratios (Supporting Information, Table S1). According to the results, increasing the support ratio from (1:1) to (1:2) enhanced conversion and lactone selectivity. However, when the catalyst-support ratio was raised to (1:3), more Lewis acid and Brnsted acid sites were present, which were responsible for the ring opening process and subsequent oxidation [69]. Therefore, the ratio (1:2) was found optimal and thus was used for further investigation.

Considering these results, the aerobic oxidation of THF was studied using ZF@γ-Al_2_O_3_ as the catalyst by varying the following factors: reaction temperature, O_2_ pressure, and reaction time. Furthermore, the optimized conditions were applied to other cyclic ether oxidations. 

#### 2.2.1. THF Oxidation 

**Figure 9:**
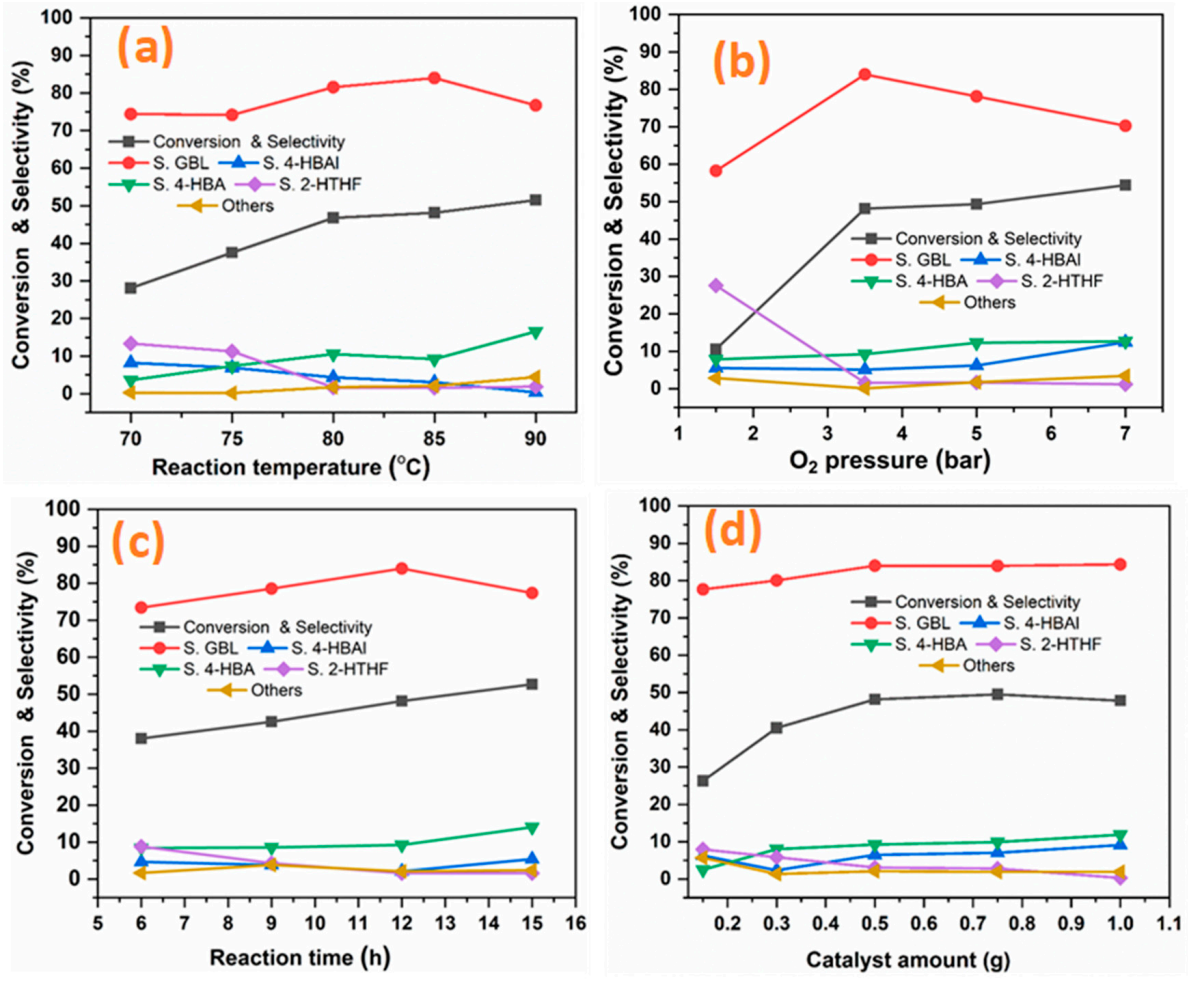
Effect of reaction parameters on the THF oxidation. (**a**) reaction temperature, (**b**) O_2_ pressure, (**c**) reaction time, and (**d**) catalyst amount. Reaction conditions (unless otherwise specified): PO_2_ = 3.5 bar, THF = 10 mL, 85 °C, 0.5 g catalyst, 12 h.

The effect of the reaction parameters on the THF oxidation is presented in Figure 9. Hence, the temperature effect was studied in the range from 70 °C to 90 °C and shown in Figure 9a. It is clear that the conversion value of GBL was remarkably dependent on the reaction temperature, as its value approximately doubled when temperature was raised from 70 °C to 85 °C. On the other hand, for the same range, the selectivity value of GBL increased from 74.39% to 83.99%. However, GBL selectivity decreased as temperatures increased to 90 °C with increased acid formation due to additional oxidation events [39]. To investigate the effect of the pressure of oxygen (PO_2_) on the rate of THF oxidation, the reaction was performed with varying PO_2_ from 1.5 to 7 bars. As seen in Figure 9b, the conversion of THF was significantly improved from 10.5% to 48.1% upon increasing PO_2_ from 1 to 3.5 bars. This increase was due to the decrease in the amount of 2-HTHF. As PO_2_ increased, GBL selectivity decreased with the formation of oxygenated products such as 4-HBAl and 4-HBA, which can be explained in terms of the fact that GBL underwent deep oxidation in the presence of excessive oxygen. Hence, these results reflect the importance of O_2_ pressure control. The reaction time effect is depicted in Figure 9c. The conversion linearly increased from 38.4 to 52.7% as reaction time increased from 5 to 15 h. On the other hand, the selectivity of GBL increased linearly by increasing reaction time up to 12 h. After 12 h, the selectivity of GBL decreased in favor of 4-HBA production. The catalyst dose was also investigated in the range of 0.15 to 1.0 g per 10 mL of substrate, as presented in Figure 9d. When the amount of the catalyst increased from 0.15 to 0.5 g, the conversion increased from 26.3 to 48.1%. This suggests that ZF@γ-Al_2_O_3_ functioned as active sites for the oxidation and, with the increase in catalyst amount, the number of active sites available for the reaction to progress increased. A gradual increase in GBL selectivity was also noticed with the increase in the catalyst up to 0.5 g, beyond which no significant change was detected. Table 3 summarizes the findings under the optimal reaction condition.

#### 2.2.2. Cyclic Ethers Oxidation

The oxidative conversion of other five- and six-membered ethers to the corresponding lactones was also investigated and presented in Table 4. According to Entry 1, saturated five-membered cyclic compounds such as 2-methyl tetrahydrofuran (2-MTHF) performed well with 40% conversion and 86% γ-valerolactone (GVL) selectively. Substrates with a six-membered ring showed lower conversion than their five-membered counterparts. This result is in line with Huckel’s theory, which states that the six-membered ring is more stable than the five-membered ring (less reactive). For instance, tetrahydropyran (THP) oxidation resulted in a low conversion of 11% but a high selectivity of 89% for δ-valerolactone (DVL) due to the absence of ring opening or excessive oxidation products. Thus, the oxidation was performed under high pressure and a long-time reaction, resulting in both improved conversion and improved selectivity of DVL production. Furthermore, due to their activity, both five- and six-membered unsaturated cyclic ethers demonstrated poor selective outcomes due to deep oxidation and polymerization reactions. Therefore, an experiment was conducted under low pressure and a shorter time, which improved selectivity results towards lactone formation (entries 2, 5 and 6).

### 2.3. Reusability

**Figure 10:**
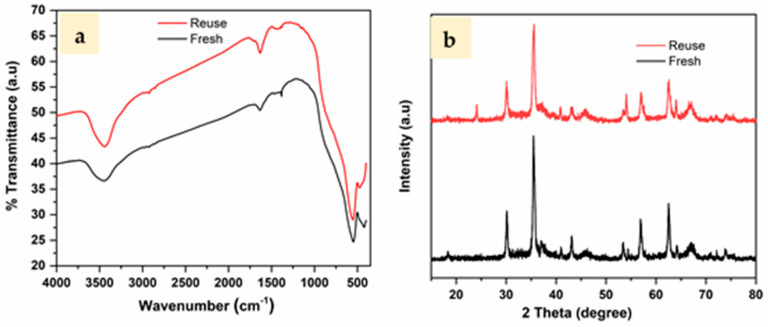
(**a**) FTIR and (**b**) XRD for fresh and reused ZF@γ-Al_2_O_3_ after the catalytic cycle.

Reusability, along with activity, is considered the most important indicator for the industrial consideration of catalysts. To reduce production costs and minimize waste generation, catalysts must be separated and recycled easily after catalytic reactions [70]. Recycling of the catalyst was performed using centrifugation, washing with aqueous ethanol (25% *v*/*v*), and finally drying at 85 °C for 24 h. FTIR and XRD techniques were used to investigate the catalyst’s stability, and the results are provided in Figure 10. As can be seen, an acceptable XRD pattern match between the fresh and reused catalysts was detected, indicating high stability of the catalyst system. However, the slight difference in the relative intensities of the peaks before and after the usage of the catalyst was due to the activation process, with the possible occupation of residues that were not removed during the reactivation stage [71]. The traced peak around 2θ of 25° was possibly for carbon residues.

**Figure 11:**
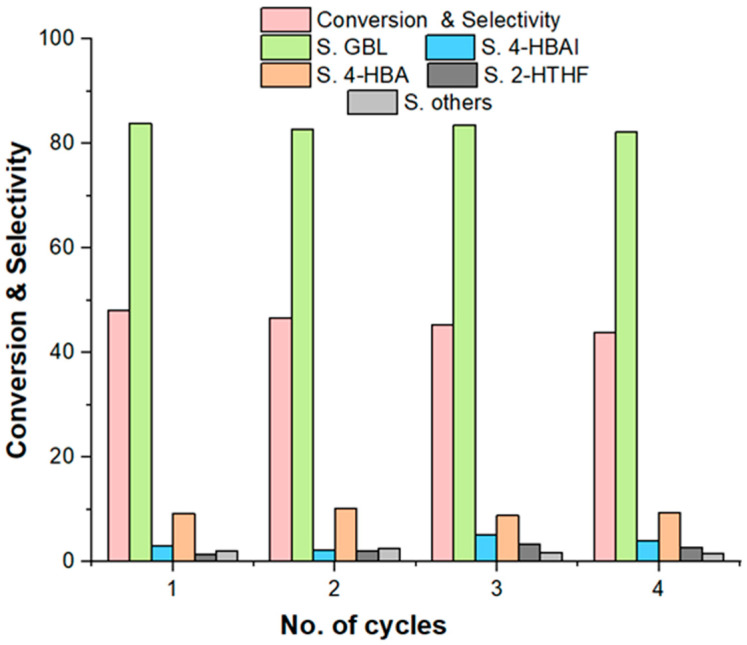
Reusability of ZF@γ-Al_2_O_3_. Reaction conditions: PO_2_ = 3.5 bar, THF = 10 mL, 85 °C, 0.5 g of catalyst, 12 h.

As can be seen in Figure 11, after four runs, a reduction in the THF conversion from 48.1% to 43.8% was observed; however, the GBL selectivity remained almost constant. A controlled leaching experiment was also performed to measure zinc and iron contents. The reaction mixture was filtered and analyzed by ICP. The results showed the absence of ions within the detection capacity of the device of 1 ppb, confirming the stability and reusability of the catalyst. These findings demonstrated the catalyst’s stability and ability to oxidize cyclic ethers to the corresponding lactones under an aerobic condition. 

## 3. Materials and Methods

### 3.1. Materials 

The organic and inorganic chemicals were used as provided without further purification and are as follows: ammonium bicarbonate (NH_4_HCO_3_), ferric nitrate nonahydrate (Fe (NO_3_)_3_·9H_2_O), and zinc acetate dihydrate (Zn(CH_3_COO)_2_.2H_2_O) from BDH chemicals, London, UK; THF (99.5%) from Fisher Scientific, Loughborough, UK; THP, 2-MTHF, 3,4-dihydro-2H-pyran, and 2,3-dihydrofuran from Sigma-Aldrich, Burlington, MA, USA; pyran, furan, and 1.4 dioxane from BDH chemicals, London, UK; and gamma-alumina (γ-Al_2_O_3_) from Sigma-Aldrich, Burlington, MA, USA.

### 3.2. Catalyst Preparation 

The gamma-alumina-supported zinc ferrite (ZF@γ-Al_2_O_3_) catalyst was prepared by a deposition–coprecipitation method. Typically, 4.04 g ferric nitrate nonahydrate, 1.1 zinc acetate dihydrate, and 1.1 g gamma-alumina (1:2 mol ratio of ZF/γ-Al_2_O_3_) were added to a 250 mL round flask containing 30 mL deionized (DI) water. The mixture was stirred at 40 °C for 1 h, and then the appropriate amount of ammonium bicarbonate in 50 mL DI was added drop-wisely. The resulting precipitate was stirred at 75 °C for a further 5 h in a closed flask. After 12 h of aging, the precipitate was filtered, washed, and dried overnight at 75 °C. Calcination at 600 °C was then performed on the catalyst precursor for 5 h.

### 3.3. Characterization of the Catalysts

Fourier transform infrared (FTIR) spectra were recorded in a PerkinElmer spectrum BX (Perkin Elmer, Waltham, MA, USA) using the KBr disk method on the range of 400 to 4000 cm^−1^. X-ray diffraction (XRD) measurements were carried out using Rigaku XtaLAB mini II benchtop X-Ray 110 crystallography system (The Woodlands, TX, USA), where Cu K∝λ = 1.5418 Å was used as the light source; the powdered sample was placed in the sample holder and exposed to X-ray radiation at room temperature, and the diffraction pattern was obtained over the 2θ range of 10° to 80° at a rate of 3°/min. X-ray photoelectron spectroscopy (XPS) was carried out using an ESCALAB 250Xi apparatus and a monochromatic Al Ka X-ray source (1486.6 eV). The specific surface area, pore volume, and average pore diameter of the catalyst were measured in Micromeritics Tristar II 3020 surface area and porosity analyzer (Micromeritics Instrument Corporation, Norcross, GA, USA). To achieve these measurements, catalysts of weights varying from 0.20 g to 0.50 g were used, and the test advancement was monitored by computer and then analyzed by the corresponding software. A JEM-2100F field emission electron microscope (JEOL, Tokyo, Japan) with an 80 kV acceleration voltage was used for transmission electron microscope (TEM) imaging; hence, drops of a prepared sample suspension were deposited on a copper grid made of lacey carbon. Scanning electron microscopy (SEM) was carried out using FESEM (JSM-7600F, JEOL, Tokyo, Japan) with an accelerating voltage of 5 kV, a beam current of 30 A, and image magnification of 30,000× for platinum-coated samples. The energy dispersive X-ray spectroscopy (EDS) profile was obtained using an X-MaxN system from Oxford instruments (Abingdon, UK). Elemental analyses were performed under inductively coupled plasma spectrometry (ICP) measurements using a Nexion 300D Spectrometer from Perkin Elmer (Waltham, MA, USA). For this, a concentrated nitric acid was used to acidify the sample. In the following steps, the acidified samples were digested in an oven at 75 °C for 24 h, cooled, filtered, and diluted as required. On a Mettler Toledo TGA/DSC Star system (Columbus, OH, USA), the thermogravimetric and differential thermal analysis (TGA/DTA) curves of the catalysts were obtained under nitrogen and at a heating rate of 10 °C min^−1^.

### 3.4. Experimental Procedure

The reactions were carried out in a 100 mL stainless steel autoclave equipped with a magnetic stirrer, manometer, and vent. Hence, the following experimental conditions were applied unless otherwise specified. A quantity of 10 mL cyclic ether substrate and 0.5 g of the catalyst were charged into the autoclave. The pressure of O_2_ was set at 3.5 bar using a regulator and mass flow meter. The reaction temperature was controlled by a heating jacket connected to a water circulator with an accuracy of ± 0.1 °C. The reactor vessel was placed under the desired temperature and continuously stirred for the predefined time. When the target time elapsed, the reactor was cooled to 10 °C and depressurized to atmospheric pressure. The used catalyst was removed by centrifugation, purified, and dried for further use. The resulting solution was analyzed in a gas chromatography (GC) instrument with a flame ionization detector (FID) using a (Rtx-5-length 30 m, ID 0.53 mm) capillary column. The identification of products was achieved occasionally by GC coupled with a mass selective detector (GC-MS). For the separation of the target compounds, the TR-5 MS-SQC capillary column (30 m length × 0.25 mm internal diameter, phase thickness 0.25 μm) was used with helium as the carrier gas (at a flow rate of 1 mL min^−1^). The total conversion, selectivity, and product yield were calculated using the following equations:

(1) $$Conversion\%=\frac{{n_{0}-n}_{r}}{n_{0}}\times 100$$

(2) $$Yield\%=\frac{n_{i}}{n_{0}}\times 100$$

(3) $$Selectivity\%=\frac{yield\left(i\right)}{conversion}\times 100$$

where $n_{0}$ is the number of the initial moles of substrate, $n_{i}$ is the number of the moles formed from the product *i* (*i* = GBL, DVL, …), and $n_{r}$ is the number of the remaining moles from substrate. 

## 4. Conclusions

In this work, the aerobic oxidation of cyclic ethers was examined over the ZF@γ-Al_2_O_-_ supported catalyst using O_2_ as an oxidizing agent under solvent-free conditions. The ZnFe_2_O_4_ was successfully incorporated on the γ-Al_2_O_3_ support, as indicated by FTIR, XRD, TEM, and BET techniques. The performance of the supported catalyst was better than that of the unsupported one in terms of conversion of the starting model material (THF) and selectivity of the desired product (GBL). The catalytic oxidation condition endorses the production of GBL as the main product. The optimized reaction conditions demonstrated that the catalytic performance was significantly influenced by temperature and amount of oxygen. The catalyst amount also greatly influenced the THF conversion, while GBL selectivity was mostly unaffected. Also, six-membered cyclic ethers were less active than five-membered cyclic ethers in terms of lactone transformation. Due to the over-oxidation and polymerization reactions that occured during the oxidation of unsaturated cyclic ethers, poor lactone selectivity was found. Controlling the reaction time and oxidant pressure are crucial for improving lactone selectivity. Thus, to achieve a high yield of the desired product, the reaction conditions (e.g., reaction time) must be carefully controlled. Hence, it is evidently proven that the applied ZF@γ-Al_2_O_3_ is promising for the selective conversion of cyclic ethers to lactones with a high product yield and catalytic activity that remained effective even after four rounds of catalysis.

## Figures and Tables

**Table 1:** BET-surface area and porosity results.

Catalyst	Total Surface Area (m^2^/g)	External Surface Area (m^2^/g)	Average Pore Volume (cm^3^/g)	Average Pore Width (nm)
ZnFe_2_O_4_	22.05	14.65	0.08	13.4
γ-Al_2_O_3_	100.55	67.35	0.22	78.0
ZF@γ-Al_2_O_3_	52.04	36.33	0.16	105.2

**Table 2:** ICP results of ZF@γ-Al_2_O_3._

Catalyst	Al (mol%)	Zn (mol%)	Fe (mol%)
ZF@γ-Al_2_O_3_	58.78	12.53	28.69

**Table 3:** Catalytic results under optimal conditions: PO_2_ = 3.5 bar, substrate (THF) = 10 mL, 0.5 g catalyst, 85 °C, 12 h.

Catalyst	Conversion (%)	Selectivity of GBL (%)
ZF@γ-Al_2_O_3_	48.1	83.9
ZnFe_2_O_4_ (ZF)	18.1	63.3
γ-Al_2_O_3_	11.7	32.6
Blank	3.2	0

**Table 4:** Cyclic ether oxidation catalyzed by ZF@γ-Al_2_O_3._

Entry	Substrate	Conversion	S. Lactone	Product
1	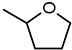	40	86	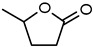
2	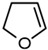	76 49 ^a^	34 62 ^a^	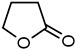
3	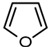	--	--	-
4	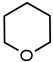	11 28 ^b^	83 89 ^b^	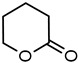
5	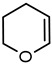	38 49 ^a^	61 72 ^a^	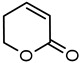
6	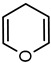	68	25	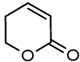
7	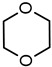	11	72	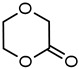

Reaction condition: substrate = 10 mL, PO_2_ = 3.5 bar, 85 °C, 0.5 g catalyst, 12 h. ^a^ Substrate = 10 mL, PO_2_ = 1.5 bar, 85 °C, 0.5 g catalyst, 6 h. ^b^ Substrate = 10 mL, PO_2_ = 7 bar, 85 °C, 0.5 g catalyst, 24 h. For byproducts, see Supporting Information Table S2.

## Data Availability

The data that support the findings of this study are available on reasonable request from the corresponding authors.

## Supplementary Materials

The following supporting information can be downloaded at https://www.mdpi.com/article/10.3390/molecules28207192/s1, Table S1: Comparison of various catalyst-support mole ratio performances in terms of conversion and selectivity. Condition: PO2 = 3.5 bar, substrate (THF) = 10 mL, 0.5 g catalyst, 85 °C, 12 h. Table S2: Products of cyclic ether oxidation reaction.
