# Supplementary material for: Fabricated Gamma-Alumina-Supported Zinc Ferrite Catalyst for Solvent-Free Aerobic Oxidation of Cyclic Ethers to Lactones
Source: Molecules. 2023 Oct 20;28(20):7192. doi: 10.3390/molecules28207192 (PMC10609177; doi:10.3390/molecules28207192)
Supplement: Supplementary file 1 [file molecules-28-07192-s001.zip › molecules-2655754-supplementary.pdf]

# Fabricated Gamma Alumina-Supported Zinc Ferrite Catalyst for Solvent-Free Aerobic Oxidation of Cyclic Ethers to Lactones

**Naaser A. Y. Abduh [1,*], Abdullah A. Al-Kahtani [1], Mabrook S. Amer [1], Tahani Saad Algarni [1], and Abdel-Basit Al-Odayni [2,*]**

## Supplementary:

**Table S1.** Comparison of various catalyst-support mole ratio performances in terms of conversion and selectivity. Condition: $PO_2$ = 3.5 bar, substrate (THF) = 10 mL, 0.5 g catalyst, 85°C, 12 h.

| Catalyst-support mole ratio | Conversion (%) | Selectivity of GBL (%) |
|---|---|---|
| 1:1 | 29.6 | 74.4 |
| 1:2 | 48.1 | 83.9 |
| 1:3 | 45.3 | 58 |

**Table S2.** Products of cyclic ethers oxidation reaction

| Entry | substrate | Product | By-products | | | |
|---|---|---|---|---|---|---|
| 1 | 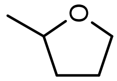 | 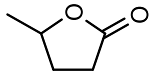 | 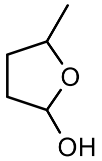 | 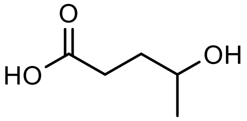 | 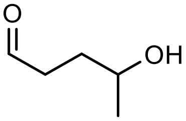 | |
| 2 | 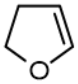 | 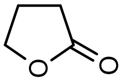 | 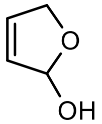 | 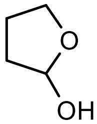 | 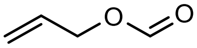 | Polymeric products |
| 3 | 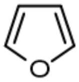 | - | | | | Polymeric products |
| 4 | 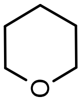 | 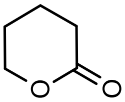 | 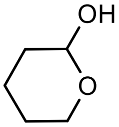 | 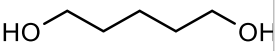 | 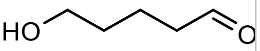 | |
| 5 | 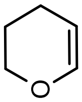 | 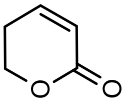 | 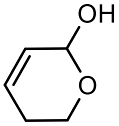 | 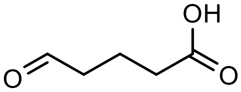 | 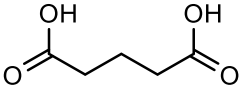 | Polymeric products |
| 6 | 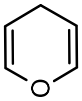 | 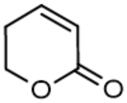 | 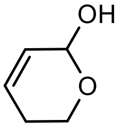 | 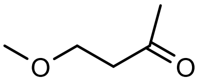 | | Polymeric products |
| 7 | 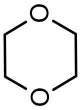 | 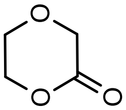 | 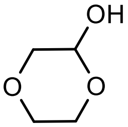 | 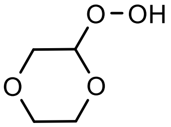 | 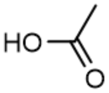 | |
